# Asymmetry of the Single Leg Jump and Lateral Shuffle Performance in Pre-Juvenile Basketball Players

**DOI:** 10.5114/jhk/196315

**Published:** 2025-04-30

**Authors:** Mengde Lyu, Zhili Chen, Shengji Deng, Ling Ding, Jia Han, Chris Bishop, Yongming Li

**Affiliations:** 1 School of Athletic Performance, Shanghai University of Sport, Shanghai, China.; 2 School of Human Sciences (Exercise and Sport Science), University of Western Australia, Perth, Australia.; 3 School of Physical Education, Shanghai University of Sport, Shanghai, China.; 4 College of Rehabilitation Sciences, Shanghai University of Medicine and Health Sciences, Shanghai, China.; 5 School of Health and Biomedical Sciences STEM College, Royal Melbourne Institute of Technology University, Melbourne, Australia.; 6 Faculty of Science and Technology, London Sport Institute, Middlesex University, London, United Kingdom.

**Keywords:** agility, inter-limb asymmetry, lateral movement

## Abstract

The aims of this study were to: 1) assess and compare the performance of lateral shuffles and lower limb jumps among pre-juvenile basketball players; and 2) calculate the asymmetry scores of these assessment methods and examine the association between their performance and existing inter-limb differences. Thirty-nine pre-juvenile basketball athletes (23 boys and 16 girls) performed the countermovement jump, the single leg countermovement jump, the single leg lateral jump, the single leg broad jump, and four shuffle tests (2 distances of 2.5 and 5 m × 2 in each direction) on separate days. Results revealed significant differences between the left and the right shuffle at distances of both 2.5 m (p < 0.05) and 5 m (p < 0.01), with inter-limb asymmetry scores ranging from 3.3 to 5.1%. There was no significant difference between the performance of the left and right legs in each single leg jump test, while the inter-limb asymmetry values ranged from 5.3 to 8.6%. Correlation analysis showed there were no correlations among the inter-limb asymmetry, shuffle asymmetry and performance, while the right single leg countermovement jump performance was significantly correlated with shuffle performance (all p < 0.001). Shuffle performance in different directions exhibited significant differences which were unrelated to inter-limb asymmetry, demonstrating the task-specific nature of asymmetry and natural variability seen in lateral movements of pre-juvenile basketball players. Practitioners are advised to use a diversified approach to assess asymmetry. These findings have implications for injury prevention and performance enhancement.

## Introduction

Basketball is a high-intensity, intermittent sport that requires athletes to frequently accelerate, decelerate, jump, and change direction (Ben Abdelkrim et al., 2007). For basketball players, possessing superior vertical (e.g., jumping) and lateral movement capabilities is crucial due to the sport's demands in multiple planes of motion (Stojanović et al., 2018). Studies indicate that jumping constitutes 0.6–2.0% of actual game time, with an average of one jump per minute, highlighting the prevalence and importance of jumping ability for basketball players (Taylor et al., 2017). Additionally, single leg jumping is a primary movement in both offensive (e.g., layups) and defensive (e.g., blocking) actions (Alemdaroğlu, 2012; Ben Abdelkrim et al., 2010). Lateral movements involve start-stop actions in the frontal plane, often performed as lateral shuffles, enabling athletes to frequently change the direction (Lyu et al., 2024; Nigg et al., 2009). Previous studies have shown that basketball players may perform over 300 lateral movements per game, accounting for 18.1–42.1% of total playing time, with an average distance of 2.28 to 4.24 m per shuffle (Taylor et al., 2017). Although research has focused on the frequency and distance of lateral movements, further investigations are needed into the specific impact this movement may have on performance. For instance, during defensive actions, the direction of lateral shuffling is unpredictable and depends on the opponent's movement patterns. Consequently, this unpredictable nature is likely to result in unequal loading on each limb and likely, the development of an inter-limb asymmetry. However, there is a notable scarcity of research examining the prevalence and relevance of inter-limb differences in lateral shuffle performance in sports such as basketball, where this movement pattern is prevalent.

Asymmetry has become popular in sport science, which may reflect structural differences in the human body and existing differences in athletic performance (Maloney, 2019; Skalski et al., 2024). Single-leg jumping, for instance, has been extensively studied to calculate inter-limb asymmetry and understand how the asymmetry impacts athletic performance(Bishop et al., 2023a; Madruga-Parera et al., 2021). Researchers use various single leg jumping tests in multiple directions, such as the single leg countermovement jump (SLCMJ), the single leg lateral jump (SLLJ), and the single leg broad jump (SLBJ), to assess inter-limb asymmetry (Bishop et al., 2023a). Findings from these tests indicate that asymmetries vary across different jump tests, underscoring the task-specific nature of limb asymmetries. Given this variability, it is recommended to employ multiple directional single leg jumping tests to achieve a more comprehensive assessment of inter-limb asymmetry (Bishop et al., 2023a).

In fact, inter-limb asymmetry is widespread among athletes engaged in various sports, such as soccer (Bishop et al., 2021), tennis (Madruga-Parera et al., 2020) and handball (Madruga-Parera et al., 2021). Inter-limb asymmetry has been shown to correlate with different movement abilities. For instance, Bishop et al. (2022b) found that asymmetry in single leg drop jump heights correlated with performance in the 505 agility test (r = 0.65, *p* < 0.01) in male soccer players. Additionally, asymmetry in SLCMJ heights is correlated with jumping (r = 0.47–0.57, *p* < 0.05) and sprinting (r = 0.49–0.59, *p* < 0.05) in female soccer players (Bishop et al., 2021). Madruga et al. (2021) discovered that asymmetry in the SLLJ was related to 180-degree change of direction (COD) ability (r = 0.39, *p* < 0.05) in youth handball players. Those studies demonstrated the significant link between inter-limb asymmetry and various sport skills. However, research has not investigated the association between both the performance and asymmetry of single leg jumping, and performance during a lateral shuffle task, especially in pre-juvenile basketball athletes. If shuffle performance correlates with inter-limb asymmetry, this relationship could help identify asymmetries and thereby improve shuffle performance. It is necessary to examine inter-limb asymmetry in basketball players and specific skills to better understand asymmetry, enhance sport performance, and prevent injury.

This study aimed to 1) assess both the shuffle and jump performance among pre-juvenile basketball players using left and right shuffle tests at two distances (i.e., 2.5 m and 5 m) and four jumping tests (i.e., the countermovement jump, the SLCMJ, the SLLJ and the SLBJ); 2) calculate the inter-limb asymmetry and asymmetry scores of shuffles, and examine the correlations among shuffle performance, jump performance, shuffle asymmetry, and inter-limb asymmetry. We hypothesized that: 1) there would be no difference between left and right shuffle performance in the 2.5-m shuffle test, but a difference would exist in the 5-m shuffle test due to the longer distance; 2) shuffle performance would be associated with jump performance and inter-limb asymmetry.

## Methods

### Participants

Thirty-nine pre-juvenile basketball players (23 boys and 16 girls, age 14.5 ± 0.5 yrs, body mass 70.0 ± 15.4 kg, body height 178.6 ± 11.1 cm, mean ± SD) were recruited in this study. The inclusion criteria were as follows: 1) both male and female athletes were elite pre-juvenile basketball players at the national level for their age group; 2) both the male and female teams ranked in the top eight in the country; 3) each teams had an average of six basketball training sessions and four strength and conditioning sessions per week. The test was conducted during the mid-season of the first half of 2024. Considering that participants were pre-juvenile, all informed consent forms were obtained from their parents. This study was approved by the Institutional Ethics Committee of the Shanghai University of Sport (protocol code: 102772024RT021; approval date: 13 April 2024).

### Design and Procedures

This study employed two sessions to evaluate shuffling and jumping performance along with asymmetry in pre-juvenile basketball players. To provide a more detailed comparison of asymmetry in shuffle performance at different distances and directions, and considering previous research indicating that the range of lateral movements was between 2.28 and 4.24 m (Taylor et al., 2017), the shuffle test included four protocols at two distances (i.e., 2.5 m and 5 m) and in two directions (i.e., left and right). Previous studies have noted the existence of task specificity in asymmetry tests and suggested that jump tests should be selected based on the specific sport of athletes (Bishop et al., 2022b, 2023a). Considering the multi-planar demands of basketball, the single leg jumping tests included the SLCMJ, the SLLJ and the SLBJ.

Testing was conducted over two sessions, spaced 72 h apart. On the first day, participants performed jump tests at the testing site, including the countermovement jump (CMJ), the SLCMJ, the SLLJ, and the SLBJ. Before the jump tests, participants completed a standardized warm-up, including jogging and dynamic stretching, followed by two practice jumps for each test. The CMJ test was performed three times, as were the single leg jump tests for each limb. The highest values from the CMJ and SLCMJ tests, and the farthest values from the SLLJ and SLBJ tests, were used for the final data analysis. The CMJ and the SLCMJ were measured using a phone app (MYJUMP2), which has been proven to be a reliable jump measuring tool (Balsalobre-Fernández et al., 2015; Bishop et al., 2022a). SLLJ and SLBJ distances were measured using a standard tape measure fixed to the floor (Madruga-Parera et al., 2021). The shuffle test was conducted on the basketball court, with areas of 2.5 m and 5 m marked on the court. Following a standardized warm-up, participants completed two practice attempts for each shuffle test. Each direction and distance of the shuffle test were performed three times, with the best score used for further data analysis.

### Measures

#### Lateral Shuffle Test

The shuffle asymmetry test was designed to include four tests at two distances and directions: 2.5-m shuffle to the left (S2.5-L), 2.5-m shuffle to the right (S2.5-R), 5-m shuffle to the left (S5-L), and 5-m shuffle to the right (S5-R). The participant stood before the starting line in their habitual shuffle posture. Two marker posts were placed at the starting and finishing lines to ensure that the participant’s front foot touched the line during movement. Participants needed to shuffle from the start line to the finish line as quickly as they could. The shuffle time was assessed using the phone app (CODTimer), which has been proven to be a reliable timing tool (Balsalobre-Fernández et al., 2019). As an example of the 5-m shuffle test, an iPhone was placed 5 m directly in front of the finish line to ensure the phone could capture the entire shuffle movement. The first frame in which the athlete's foot left the ground (start of the shuffle) and the frame in which the athlete's foot crossed the marker post (end of the shuffle) were selected for calculating shuffle performance. The performance of the 2.5-m and 5-m shuffles in both directions (left and right) was summed to form the final composite performance for the 2.5-m (S2.5) and 5-m (S5) shuffles.

#### Countermovement Jump Test

Participants stood with their hands on their hips, performed a countermovement to a self-selected depth, and then jumped as high as possible when ready. Three trials were conducted with a 60-s rest interval between each trial. Value obtained in the highest jump was used for subsequent data analysis.

#### Single Leg Countermovement Jump Test

Participants stood on one leg with their hands on their hips, then performed a countermovement to a self-selected depth and jumped as high as possible using the standing leg when ready. During the jump, additional swinging of the non-jumping leg was not permitted. The non-jumping leg was slightly bent at the knee, with the foot hovering near the ankle of the jumping leg. Three trials of the SLCMJ were conducted for each leg, with a 60-s rest interval between each trial. The jump test was invalidated if the jump movement was incorrect. Value of the highest jump on each leg was used for subsequent data analysis (Madruga-Parera et al., 2021).

#### Single Leg Lateral Jump Test

Participants started the jump from the 0-cm mark with one leg, performed a countermovement to a self-selected depth, and then jumped laterally as far as possible. When starting from the left leg, participants were instructed to keep both hands on their hips throughout the entire test and jump as far to the right as possible. Participants landed using both legs to increase stability, held the landing position for 2 s, and then measured the distance from the outer edge of the landing foot (the part closest to the 0-cm mark). Three trials were conducted for each leg, with a 60-s rest interval in between, and the trial with the longest jump on each leg was used for data analysis (Madruga-Parera et al., 2020).

#### Single Leg Broad Jump Test

Participants stood on one leg at the 0-cm mark, performed a countermovement to a self-selected depth, and then jumped forward as far as possible while keeping their hands on their hips throughout the jump. Participants were instructed to hold the landing for 2 s, and the distance was measured from the heel of the jumping foot. Three trials were conducted for each leg, with a 60-s rest interval between each trial. The furthest jump on each leg was used for data analysis (Madruga-Parera et al., 2020).

### Statistical Analysis

The descriptive data are presented as means ± standard deviations (SDs). Normality of the data distribution and homogeneity of variance were assessed using the Shapiro-Wilk and Levene’s methods, respectively. Reliability was assessed using a two-way random ICC with absolute agreement and 95% confidence intervals, typical error of the measurement (TEM), and the coefficient of variation (CV). The magnitude of the CV was interpreted as follows: poor (>10%), moderate (5–10%) or good (<5%) (Banyard et al., 2017), and the intraclass correlation coefficient (ICC) was interpreted in line with Koo and Li (2016), where values excellent were > 0.9, good 0.75–0.9, moderate 0.5–0.75 and poor < 0.5. Asymmetries were calculated for all tasks defining the dominant (D) (the limb with the better score) and non-dominant (ND) limbs, using the following formula: asymmetry index = (D – ND) / D * 100% (Bishop et al., 2023a). Paired samples *t*-tests were used to compare the difference among shuffle performances at different distances and directions. Cohen’s *d* effect sizes (ES) with 95% confidence intervals were calculated for pairwise comparisons which were computed as the mean difference divided by the pooled standard deviation and defined as: small (< 0.2), moderate (0.2–0.5), and large (> 0.8) mean differences (Cohen, 2013). Statistical significance was set at *p* < 0.05. Pearson’s r correlations and Spearman’s ρ correlations were employed to examine the relationship between all results of performance and inter-limb asymmetry data that followed and did not follow a normal distribution, respectively. The magnitude of the correlation between test indicators was categorized as follows: trivial (≤ 0.1), small (0.1–0.3), moderate (0.3–0.5), large (0.5–0.7), very large (0.7–0.9) and almost perfect (0.9–1.0) (Hopkins et al., 2009). Bonferroni corrections were applied to all correlations to account for type II error rate, resulting in statistical significance being set at *p* < 0.00167 when comparing the correlations between asymmetry percentages and shuffle performance, and being set at *p* < 0.00119 when comparing the correlations between jump and shuffle performance. All statistical analyses were conducted using IBM SPSS software version 26.0 (IBM, Armonk, New York, USA).

## Results

**Figure 1:**
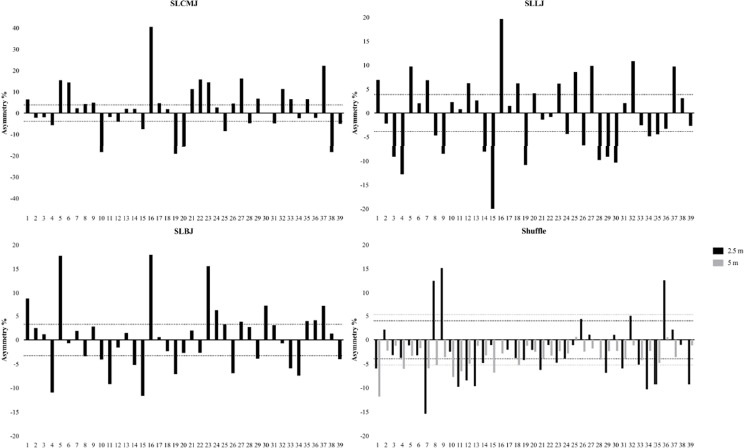
Individual inter-limb asymmetry data for jump height and shuffle speed. Above 0 means the asymmetry favors the right limb or direction, and below 0 means asymmetry favors the left limb or direction. Dotted lines represent the group threshold calculated from the pooled CV of the right and left limbs or direction test scores (SLCMJ = 3.85%, SLLJ = 3.85%, SLBJ = 3.25%, S2.5 = 3.95%, S5 = 5.25%). Abbreviation: SLCMJ, single leg countermovement jump; CMJ, countermovement jump; SLLJ, single leg lateral jump; SLBJ, single leg broad jump; CV, coefficient of variation

Table 1 shows the performance, asymmetry, and reliability data. The ICC values were reported good to excellent for all tests (≥ 0.90). CV values were acceptable for all tests (< 10%). The shuffle and jump performance data followed a normal distribution, whereas the asymmetry data did not conform to a normal distribution. There were no significant differences observed among the performances of single leg jumping on the left and right legs (all *p* > 0.05). Significant differences were found between S2.5-L and S2.5-R, and between S5-L and S5-R (*p* < 0.01), with leftward shuffle performed better than rightward shuffle. Figure 1 shows the individual asymmetry scores with directional differences.

**Table 1:** Jump and shuffle performance, asymmetry scores, and test reliability data.

Test	Mean ± SD	Effect Size (95% CI)	Asymmetry (%)	TEM (95% CI)	CV (%)	ICC (95% CI)
SLCMJ-L	16.48 ± 3.29	−0.13 (−0.57–0.32)	8.6 ± 8.0	0.26 (0.20–0.32)	2.2 (0.3–3.9)	0.98 (0.98–0.99)
SLCMJ-R	16.87 ± 2.91			0.30 (0.24–0.36)	2.4 (0.2–3.8)	0.98 (0.98–0.99)
CMJ	32.37 ± 4.75			0.74 (0.28–1.62)	3.9 (1.3–10.2)	0.98 (0.97–0.99)
SLLJ-L	134.46 ± 21.09	0.04 (−0.40–0.49)	6.6 ± 4.7	2.10 (1.60–2.50)	1.9 (0.2–3.8)	0.99 (0.98–0.99)
SLLJ-R	133.59 ± 19.01			1.99 (1.58–2.39)	2.1 (0.3–3.9)	0.98 (0.96–0.99)
SLBJ-L	151.97 ± 20.71	0.04 (–0.48–0.41)	5.3 ± 4.5	2.21 (1.77–2.66)	1.8 (0.3–3.3)	0.98 (0.97–0.99)
SLBJ-R	152.64 ± 16.95			2.19 (1.69–2.69)	2.0 (0.4–3.2)	0.97 (0.95–0.98)
S2.5-L	2.80 ± 0.29^*^	0.27 (−0.17–0.72)	5.1 ± 4.2	0.02 (0.01–0.02)	2.4 (0.5–4.0)	0.93 (0.88–0.96)
S2.5-R	2.73 ± 0.22			0.02 (0.01–0.02)	2.2 (0.5–3.9)	0.90 (0.83–0.94)
S2.5	5.53 ± 0.47			0.02 (0.01–0.04)	1.5 (0.3–3.3)	0.96 (0.94–0.98)
S5-L	3.16 ± 0.23^**^	0.52 (0.07–0.97)	3.3 ± 2.5	0.03 (0.02–0.03)	2.0 (0.5–5.2)	0.95 (0.91–0.97)
S5-R	3.05 ± 0.19			0.03 (0.02–0.04)	2.0 (0.3–5.3)	0.92 (0.87–0.96)
S5	6.21 ± 0.40			0.03 (0.01–0.09)	1.8 (0.3–5.5)	0.90 (0.84–0.95)

Abbreviation: SLCMJ, single leg countermovement jump; CMJ, countermovement jump; SLLJ, single leg lateral jump; SLBJ, single leg broad jump; S2.5, shuffle test at 2.5 m; S5, shuffle test at 5 m; L, left direction; R, right direction; ^*^ significantly different compared to the test on the right (p < 0.05); ^**^ significantly different compared to the test on the right (p < 0.001)

Table 2 shows no meaningful correlations between inter-limb asymmetry scores and shuffle performance (all *p* > 0.001), while only 5-m shuffle asymmetry was correlated with shuffle performance (*p* < 0.001). Table 3 displays the correlations between jump performance and shuffle performance, with the CMJ height exhibiting the highest correlation with shuffle performance (r = 0.67). In the single leg jump tests, SLCMJ performance also showed a large correlation with shuffling (r = 0.60). For the SLCMJ and SLLJ tests, whether at 2.5 m or 5 m, shuffle performance was more strongly associated with the jumping performance of the right leg.

**Table 2:** Correlations between asymmetry percentages and shuffle performance.

	S2.5	S5	S2.5-L	S2.5-R	S5-L	S5-R
SLCMJ %	–0.15	–0.04	–0.12	–0.07	–0.04	0.02
SLLJ %	–0.01	–0.06	–0.12	0.10	–0.04	–0.05
SLBJ %	0.04	–0.14	0.02	–0.04	–0.10	–0.21
S2.5 %	0.26	0.10	0.22	0.05	0.14	0.06
S5 %	0.50^*^	0.37	0.40	0.46	0.49	0.21

Abbreviation: SLCMJ, single leg countermovement jump; CMJ, countermovement jump; SLLJ, single leg lateral jump; SLBJ, single leg broad jump; S2.5, shuffle test at 2.5 m; S5, shuffle test at 5 m; L, left direction; R, right direction; ^*^ statistically significant after Bonferroni correction (p < 0.00167)

**Table 3:** Correlations between jump and shuffle performance.

	CMJ	SLCMJ-L	SLCMJ-R	SLLJ-L	SLLJ-R	SLBJ-L	SLBJ-R
S2.5	0.40	0.46	0.52^*^	0.38	0.45	0.42	0.32
S5	0.67^*^	0.56^*^	0.60^*^	0.46	0.54^*^	0.48	0.57^*^
S2.5-L	0.40	0.48	0.49^*^	0.40	0.50^*^	0.45	0.32
S2.5-R	0.40	0.44	0.56^*^	0.33	0.44	0.38	0.33
S5L	0.64^*^	0.55^*^	0.59^*^	0.45	0.52^*^	0.48	0.55^*^
S5R	0.66^*^	0.53^*^	0.60^*^	0.45	0.56^*^	0.46	0.57^*^

Abbreviation: SLCMJ, single leg countermovement jump; CMJ, countermovement jump; SLLJ, single leg lateral jump; SLBJ, single leg broad jump; S2.5, shuffle test at 2.5 m; S5, shuffle test at 5 m; L, left direction; R, right direction; ^*^ statistically significant after Bonferroni correction (p < 0.00119)

## Discussion

The aims of this study were to 1) assess shuffle and jump performance among pre-juvenile basketball players, and 2) calculate inter-limb and shuffle asymmetry, and examine the correlations among shuffle performance, shuffle asymmetry, jump performance, and inter-limb asymmetry. Our results indicated that 1) significant differences in shuffle performance were observed between directions in the 2.5-m and 5-m tests, and 2) inter-limb asymmetry was not correlated with shuffle performance, but the right leg's jump performance appeared to be the most significant factor correlating with shuffle performance.

The observed differences in shuffle performance aligned with our expectations, as similar studies have shown lateral movement differences in tennis players (Salonikidis and Zafeiridis, 2008). The results indicate that shuffle speed was faster to the left than the right for both distances, and this asymmetry became more pronounced with increased distance. Despite the observed shuffle asymmetry, no correlation was found between shuffle asymmetry and inter-limb asymmetry (all *p* > 0.05). This indicates that unilateral jump test asymmetry reflects inter-limb differences, but does not explain performance differences in shuffles.

Additionally, weak correlations between unilateral jump test asymmetry and shuffle performance (all *p* > 0.05) may indicate that looking for associations with inter-limb differences is not worth practitioner time because of the inherent noise that accompanies ratio values (Bishop et al., 2023b). In contrast, the raw jump data (Table 3) show much stronger associations. This aligns with previous research which indicated that unilateral jump asymmetry was not correlated with the 505 and T-tests (Lockie et al., 2014). These findings suggest that shuffle and other movement tests may involve more complex neuromuscular control and coordination skills that may not be captured by single leg jumping tests. Therefore, it is crucial to consider a variety of physical tests to enhance the athlete’s motor abilities and reduce the risk of injury. Additionally, we found that shuffle asymmetry was not related to inter-limb asymmetry, highlighting the task-specific nature of asymmetry (Bishop et al., 2023a). We believe this may be due to the natural variability present in lateral movements. This variability could arise from individual physiological differences, training backgrounds or even the specific techniques and strategies used during routine training (Bishop et al., 2018, 2023a). For example, different coaches might teach slightly varied techniques for lateral shuffle, and athletes in different training environments might develop distinct movement habits, all of which can be reflected in their performance of lateral movements. Given the task-specific nature of asymmetry and the natural variability observed, practitioners should adopt a diversified approach in assessing and training athletes. Therefore, it is recommended to introduce more specialized assessment methods and personalized training programs to identify and optimize these asymmetries and variabilities, thereby enhancing performance and reducing the risk of injury (Afonso et al., 2022).

The correlation between shuffle performance and jump performance showed that all jump performances were related to shuffle performance, with the CMJ displaying the highest correlation (r = 0.67, *p* < 0.001), confirming the close relationship between shuffle performance and lower limb neuromuscular capability. The SLCMJ had the highest correlation with shuffle performance (r = 0.60, *p* < 0.00119) in single leg jumping tests, highlighting the sensitivity of the SLCMJ test in assessing lower limb neuromuscular capability (Bishop et al., 2021). Notably, in both SLCMJ and SLLJ results, the right leg showed stronger correlations with shuffle performance than the left leg, regardless of the shuffle direction. This is probably because the dominant limb for the majority of the sample (37 out of 39) was the same. This insight could be vital for designing tailored training programs that specifically focus on enhancing right leg strength and coordination.

The strong correlation between jump performance, particularly in countermovement and lateral jumps, and shuffle performance underscores the importance of focusing training on both unilateral strength and coordination (Pérez-Ifrán et al., 2023). This approach may potentially enhance overall athletic performance and reduce the risk of injury. Furthermore, the difference in shuffle speed between the left and right directions suggests an underlying dominance pattern. This pattern may not align with traditional definitions of leg dominance, but reflects performance in sport-specific movements (Haaland and Hoff, 2003). This highlights the importance of the understanding of the dominant leg in athletic contexts, which can differ from common biomechanical assessments. Previous research comparing various test asymmetries has typically contrasted the D (better performing) side with the ND (poorer performing) side (Ding et al., 2024); only few studies reported information about left-right differences (Bishop et al., 2022a). Previous studies also suggest that the definition of the D leg is not clear. For example, Miyaguchi and Demura (2010) indicated that when selecting a leg for a single leg stance to maintain balance, the left leg is predominantly chosen; conversely, when selecting a leg to kick a ball, the right leg is more frequently preferred. In training practice, reporting performance differences between the left and right sides may be more applicable for training arrangements.

Basketball coaches should pay attention to players' shuffle asymmetry to specifically enhance their agility and movement skills. This is crucial for coaches when arranging tactics on the court, especially in balancing athlete performance during defense. In pre-juvenile basketball players, inter-limb asymmetry is a significant and common issue. This asymmetry often manifests in strength, power, and motor control (Kiesel et al., 2014), and also impacts performance and increases injury risk (Afonso et al., 2022). Therefore, training programs should focus on balancing the development of strength and coordination in both limbs (Cadens et al., 2023; Zhao et al., 2023). For example, incorporating bilateral exercises and contralateral strengthening exercises can help reduce asymmetry (Bettariga et al., 2022), thereby improving overall performance and reducing injury risk. Identifying and correcting these asymmetries is crucial for the long-term health and athletic development of adolescent basketball players (Afonso et al., 2022).

There were some limitations of this study. First, we only tested shuffles in the lateral direction, while in actual training and competition, shuffles can occur in various directions and are often combined with COD in basketball games, but we did not design tests for asymmetry in shuffles combined with COD. Future research should focus on these two points to better understand inter-limb asymmetry in basketball players. Second, the training background of participants prior to the evaluation tests may have lacked control. Although all athletes were from the same training center, different training histories may have influenced the performance outcomes, thereby affecting the comparability of results. Future research should aim to control for participants' training backgrounds or provide a detailed description of their previous training regimens to minimize the impact of this confounding factor on the study results. Another limitation of this study is the small and non-representative sample size. Since the study sample was drawn from a specific age group, gender, and training background, the findings may not fully reflect the situation of a broader population. In addition, the small sample size may reduce the ability to detect actual effects or differences. Future research should consider expanding the sample size and including more participants with diverse characteristics to improve the generalizability and representativeness of the results. However, this research reveals asymmetries and significant directional differences in shuffle performance among basketball players, and it also emphasizes the task-specific nature of inter-limb asymmetries. To our knowledge, this is the first study to evaluate shuffle performance and analyze asymmetry in basketball players. Our findings can help coaches and practitioners recognize the necessity of evaluating and training shuffle performance and inter-limb asymmetry in basketball players, which would facilitate more targeted training for basketball skills and physical conditioning, especially in terms of improving defensive abilities.

## Conclusions

In conclusion, the present study demonstrated that there were observable asymmetry scores in shuffle performance among pre-juvenile basketball players at distances of 2.5 m and 5 m, which became more pronounced with increased distance. Although unilateral jump asymmetry did not correlate with shuffle asymmetry or shuffle performance, performance of the right leg in jump tests showed a stronger influence on shuffle performance across both tested distances. These findings underscore the complexity of lateral movement and its dependency on lower limb neuromuscular capabilities.
